# Undernutrition and associated factors among lactating mothers in rural Yilmana Densa District, Northwest Ethiopia: A community‐based cross‐sectional study

**DOI:** 10.1002/fsn3.3176

**Published:** 2022-12-12

**Authors:** Biruk Yazie Wubetie, Tigist Kefale Mekonen

**Affiliations:** ^1^ College of Agriculture and Environmental Science Bahir Dar University Bahir Dar Ethiopia; ^2^ College of Agriculture and Natural resources Debre Markos University Debre Markos Ethiopia

**Keywords:** body mass index, lactating mother, rural Yilmana Densa District, undernutrition

## Abstract

Undernutrition is continued to be significant public health problem worldwide. The extra calories and nutrients required to support breastfeeding make lactating mothers at higher risk of malnutrition than general population. Undernourished lactating mothers have also been found to influence both the quantity and quality of breast milk and then the nutritional and health status of their offspring. Different evidence showed that undernutrition among lactating mothers is a serious public health problem in Ethiopia in which one of every four lactate mothers are undernourished. Despite this fact, the prevalence of undernutrition among lactating women in Ethiopian was not well investigated and very limited number of studies are conducted. This study aims to assess the prevalence of undernutrition and associated factors among lactating mothers in rural Yilmana Densa District, Northwest Ethiopia. A community‐based cross‐sectional study was conducted among 428 lactating mothers. The data were collected by using interviewers administered structured questionnaire; and also, anthropometric measurements were taken from the study participants. Binary logistic regression model was undertaken to identify significantly associated factors with undernutrition. The prevalence of undernutrition among lactating mothers was 22.6%. Household income, food security status, dietary diversity score, number of meals, potable water source, and latrine facility were found to be significantly associated with undernourishment of lactating mothers. A significant proportion of lactating mothers in the district suffered from undernutrition and hence, to improve nutritional status of lactating mothers, strategies should focus on nutrition counseling, advancing diversified production and consumption, improvement of access to potable water and latrine, as well as effective household food security interventions.

## Graphical abstract

This study found that the prevalence of undernutrition among lactating mothers is high, that is, more than one‐fifth of lactating mothers are undernourished. Moreover, different factors were significantly associated with undernutrition among lactating mothers in the study area.

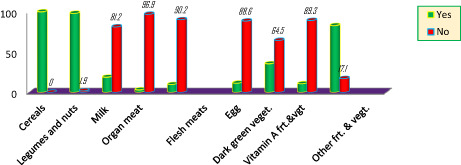

## 1 BACKGROUND

Malnutrition refers to the problem related to nutrition and includes both undernutrition and overnutrition. However, in most parts of the world, the most common form of malnutrition is undernutrition which is due to inadequate protein, energy, and micronutrient intake; and low‐income countries have suffered from high levels of undernutrition (Victora et al., 2008). According to a WHO report, the nutritional requirements increase when a woman is breastfeeding and hence women must eat a sufficient quantity of food during this period (World Health Organization (WHO), 2009). Therefore, undernutrition during lactation is one of the determinants of women's health and the health of the next generation. The nutritional status of lactating mothers is an important public health issue since their nutrition status may influence both the quantity and quality of nutrient concentration of breast milk, and also maintaining the nutrients in the breast milk further depletes their own body stores (Nakamori et al., 2009). Lactating mother is not only living for herself but also for the infant, family, and society and she carries great responsibility in the family (Hundera et al., 2015). Chronic undernutrition among women is a major risk factor for adverse birth outcomes (CSA, 2005). To support lactation and maintain maternal reserves, mothers need to eat about 500 additional kilocalories every day since the nutritional requirements are greater in lactation than in pregnancy as nursing mothers produce 0.7 to 0.8 L/d milk (Alemayehu et al., 2015; Roba et al., 2015).

In developing countries, especially in Sub‐Saharan Africa, including Ethiopia, there is a high burden of undernutrition problem among lactating mothers (Abeya et al., 2018). Even, the prevalence of undernutrition is higher in Ethiopia than in other sub‐Saharan African countries based on the most recent DHS survey conducted (Bitew & Telake, 2010). Therefore, undernutrition among lactating mothers is a serious public health problem in Ethiopia; and it is evident that one of every four lactate mothers in Ethiopia is undernourished (CSA, 2016). And also, according to different pocket studies conducted in Oromia, Amhara, Tigray, and Southern Nations, about one‐fifth of lactating mothers were underweight (Berhanu et al., 2017; Hundera et al., 2015; Ismael et al., 2017). This indicates that the prevalence of undernutrition was significant, and unacceptably high numbers of lactating mothers are suffering from undernutrition (Hundera et al., 2015).

In low‐income countries including Ethiopia, 20%–25% of women are underweight (Chaparro et al., 2014; Hundera et al., 2015). Household food insecurity, low dietary diversity score, low level of educational status, small landholding size, poor ANC visits, inadequate household environment like water and toilet facility, and high burden of reproduction were reported as determinants of maternal undernutrition in Ethiopia (Berhanu et al., 2017; Black et al., 2008; Chaparro et al., 2014; Hundera et al., 2015). In addition, lactating mothers are considered a nutritionally vulnerable group due to frequent pregnancy, caring, and nurturing of the family as well as gender‐based workload which in turn leads to high maternal mortality (Engidaw et al., 2019).

In order to address this nutritional problem, the government of Ethiopia has developed a National Nutrition Strategy with a long‐term program lasting from 2008 to 2013 and divided into two phases based on a thorough assessment of problems and issues in implementation (Alemayehu et al., 2015). Hence, the Ministry of Health of Ethiopia in collaboration with partner organizations developed the National Nutrition Program (NNP) to implement the strategy with the aim of reducing the magnitude of malnutrition among under 5 children, pregnant, and lactating mothers (Hundera et al., 2015). Moreover, to mitigate underweight, the government of Ethiopia has tried to capacitate the Health Extension Workers and all other health workers by providing preservice and in‐service trainings. Additionally, awareness rising was continuously given to pregnant and lactating mothers using Female Development Army, and also nutritional support is being given to those pregnant and lactating mothers exposed to underweight after screening in the selected vulnerable places of a country. Despite these efforts, its effect in improving the nutritional status of vulnerable groups including lactating mothers is not as such as expected and the situation continues to prevail. Moreover, as per our search, there is a limited study conducted in Amhara region including the study area. Even those studies did not incorporate valuable factors, mainly food security status and dietary diversity, because mostly the data are extracted from other datasets like DHS Dataset. Therefore, this study was conducted to assess the prevalence of undernutrition and associated factors among lactating mothers through community‐based cross‐sectional study design.

### 1.1 Conceptual framework

**FIGURE 1:**
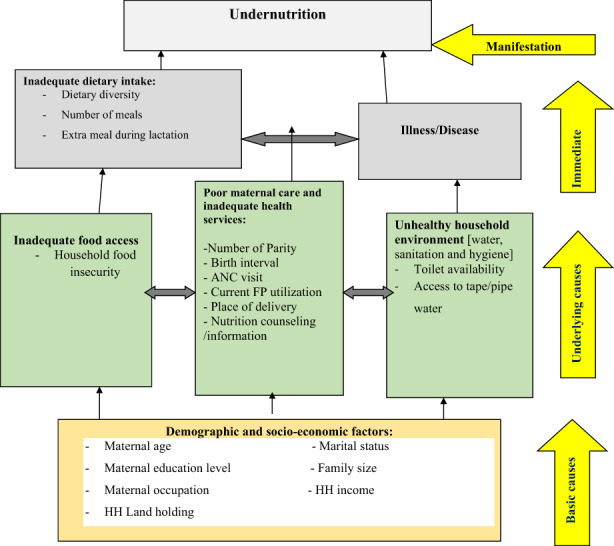
Conceptual framework for undernutrition and associated factors among lactating mothers. *Source*: Modified from UNICEF, 2001.

As shown in Figure 1, the conceptual framework showed that nutritional status of lactating mothers is determined by immediate, underlying, and basic factors.

## 2 METHODS AND MATERIALS

### 2.1 Study design, area, and period

Community‐based cross‐sectional study design was conducted in Northwest Ethiopia from July 1, to July 30, 2019. The study area is located 600 km from Addis Ababa, the capital city of Ethiopia, and 42 km from Bahir Dar, the capital city of the Amhara region. The district has a total of 24 kebeles, 11 health centers, and 25 health posts. According to the population projection for 2016/17, the population of the district was 77,195 males and 79,772 females, totally 156,967 in 36,504 households.

### 2.2 Population

For this study, the source population was all lactating mothers with 0–24 months aged child residing in the rural Yilmana Densa district. All lactating mothers with 0–24 months aged child and who lived in the selected kebeles of rural Yilmana Densa District being available during the study period were the study population.

### 2.3 Inclusion and exclusion criteria

Lactating mothers who lived in the area for 6 or more months with children 0–24 months of age during the study period were included in the study and none of the respondents were excluded from the study.

### 2.4 Sample size determination and sampling procedures

The required sample size of this study was calculated using both single‐population proportion and double‐population proportion.

For specific objective 1: the sample size for this study was calculated by using a single‐population proportion formula by considering 5% marginal error and 95% CI and design effect of 1.5 (since only two‐stage sampling is employed in sampling procedure) with none response rate of 10%.

$$n=\frac{\;{\left(Z_{\alpha /2}\right)}^{2*}\;P\left(q\right)}{d^{2}}=\frac{{\left(1.96\right)}^{2*}P\left(1-P\right)}{{\left(0.05\right)}^{2}}$$

where *n* = the required sample size; *Z*_*α*/2_ = 95% confidence (1.96); *P* = prevalence of the outcome of interest; *d* = desired precision (5%) margin of error (0.05).

Therefore, using the prevalence of undernutrition among lactating mothers in the rural Ambo district which is 21.5% (Zerihun et al., 2016), the sample size was as follows:

$$n_{1}={\left(1.96\right)}^{2}0.215\left(1-0.215\right)/{\left(0.05\right)}^{2}=259.$$

The sample size for the second objective was calculated using double‐population proportion formula based on the following assumptions:

$$\frac{{\left[z_{\frac{\alpha }{2}}\sqrt{\left(1+\frac{1}{r}\right)p\left(1-p\right)}+z_{\beta \sqrt{p1\left(1-p\right)+\left(\frac{p2\left(1-p2\right)}{r}\right)}}\right]}^{2}}{{\left[p1-p2\right]}^{2}}$$

where $P=\frac{p1+\mathrm{rp}2}{1+r}$; *p*1 = prevalence of undernutrition among exposed/diseased with exposed/for cases; *p*2 = prevalence of under nutrition among nonexposed/diseased but not exposed/for controls; *r* = control‐to‐case ratio = 1:1.

Therefore, considering the above assumptions required, sample size for the second objective was calculated for some associated factors as follows:

**Table:** 

No	Major associated factors	Confidence level (%)	Power (%)	Prevalence of undernutrition among exposed/diseased with exposed/for cases (%)	Prevalence of undernutrition among nonexposed/diseased but not exposed/for controls (%)	Control‐to‐case ratio	Odds ratio	Nonresponse rate	Required sample size
1	Extra meal	95	80	26.4	4	1:1	20.6	10	96
2	Food security	95	80	29.02	14	1:1	3.04	10	208
3	Education level	95	80	22.9	10.5	1:1	2.31	10	254

Finally, 259 lactating mothers from the first specific objective were considered as study participants since it gives larger sample size; but by considering design effect of 1.5 and 10% of the sample size, the final sample size was 428 lactating mothers.

In this study, a two‐stage sampling technique was used to select study participants. First, from the 24 rural kebeles in the district, 7 kebeles were selected randomly by using a lottery method. All households in the kebeles with lactating women were identified with the help of health extension workers. Then, systematic random sampling technique was employed to select households, the first lactating mother was selected using lottery method, and lastly, a total of 428 study participants were included by proportionally allocating to each kebeles based on their number of lactating mothers. In households where there is more than one lactating mother, lottery method again was used to select one study participant.

### 2.5 Variables of the study

#### 2.5.1 Dependent variable

Undernutrition among lactating mothers.

#### 2.5.2 Independent variables

- Basic factors like maternal age, marital status, maternal educational level, family size, occupation, household income, and household landholding.
- Underlying factors like food security status, place of delivery, birth interval, number of parity, current family planning utilization, ANC visit, nutrition counseling/information, accessibility of toilet, and potable water source.
- Immediate factors such as dietary diversity, number of meals, intake of extra meals, and illness/sickness were considered as independent variables.

### 2.6 Operational definitions

- **Lactating mothers:** A woman who has 0‐ to 24‐month‐old breastfed child (in Ethiopian context).
- **Undernutrition:** Lactating mothers whose body weight is too low, i.e., BMI < 18.5 kg/m^2^.
- **Women Dietary Diversity score**: It is a qualitative measure of food consumption that reflects mothers' access to a variety of foods and is also a proxy measure of nutrient adequacy of the diet which is measured among nine food groups (Haddad et al., 2015).
- **Food security**: Those lactating mothers' households respond “yes” to one of the nine HFIAS generic questions [except for question number 1 which is yes and it rarely occurs] in the Household Food Insecurity Access Scale (HFIAS) measurement tool was considered as food insecure otherwise food secured

### 2.7 Data collection tools and procedures

Data were collected through interviewers administering structured questionnaires by face‐to‐face interview to lactating mothers at each selected kebeles. The questionnaire mainly focuses on questions related to basic, underlying, and immediate factors of undernutrition and anthropometric measurements. The data were collected by recruiting seven health extension workers and two supervisors.

#### 2.7.1 Dietary diversity measurements

Women's Dietary Diversity Score of subjects was calculated by adding their responses for nine food groups consumed based on a 24‐h recall period.

#### 2.7.2 Food security measurement

Food security status was assessed using Household Food Insecurity Access Scale (HFIAS), a tool validated in other developing countries. The HFIAS has nine generic questions asking household's last month experience about three domains of food insecurity: feeling anxiety and uncertainty of food supply, insufficient quality of food, and insufficient quantity food intake and its physical consequences. Participating lactating mothers were categorized into two categories as food secure and food insecure according to the recommendation in the HFIAS manual (Chauhan & Kumar, 2016).

#### 2.7.3 Anthropometric measurements

The nutritional status of the mothers was assessed by using the body mass index. So, measures of height in centimeters (to the nearest 0.1 cm) and weight in kilograms (to the nearest 0.1 kg) for every mother were taken using a weighing scale with an attached height meter (Seca). Measurements of height and weight were done with no shoes and with light closing as much as possible. Three measurements at a time were taken for each mother, and in the final analysis, the average of three measurements was taken (Cogill, 2003). Mid‐upper arm circumference was also measured by using nonstretchable MUAC tape.

### 2.8 Data quality assurance

The quality of data was assured through careful design of the questionnaire and data collection procedure. The questionnaires were prepared in English language and then translated into the local language (Amharic) and it was translated back to English to check for its consistency. Training on the data collection procedures and ethical issues was given to the data collectors and supervisors. A pretest was held on 5% of lactating mothers prior to data collection in the area other than the selected districts and then the data were checked by supervisors daily for its completeness and consistency. And also, the principal investigator monitored the overall quality of data collection, and carefully entered and thoroughly cleaned the data before the analysis.

### 2.9 Data processing and analysis

All relevant data were gathered and raw data were cleaned, coded, and entered into Epi‐Data Version 3.1 software. Then, the data were analyzed with descriptive statistics including mean with standard deviation, proportions, and frequency distribution tables by using SPSS Version 22. In addition, logistic regression model was used to analyze the data. First, bivariable logistic regression was used to assess the crude association between dependent and independent variables; and the independent variables with p‐value less than 0.25 during the bivariable analysis were selected as candidate for multivariable analysis to identify key significant factors that have been associated with outcome variables. Finally, significant association of independent variables with outcome variables was declared by using adjusted odds ratio with 95% confidence interval, and variables with a *p*‐value of less than or equal to .05 were taken as statistically significant by employing binary logistic regression.

## 3 RESULTS

### 3.1 Demographic and socioeconomic characteristics of the respondents

As shown in Table 1, 420 lactating mothers with response rate of 98.13% were assessed. The result of this study showed that the mean age of lactating mothers was 29.14 (±6.50) years with 10.9 **±** (6.869‐month‐old child). The mean family size was also 4.2 **±** (1.322), and almost 85% of respondents had family size less than or equals to five. The result of this study in Table 1 also shows that all of the respondents were from the ethnicity of Amhara and 98.6% of them were Orthodox Christians. In addition, 44.3%, 87.9%, and 98% of the respondents cannot read and write, farmers, and married, respectively, with the average landholding size of 1.16 **±** (.4691) hectares. The median of household monthly income was also found to be 1600 Ethiopian birr.

**TABLE 1:** Demographic and socioeconomic characteristics of the respondents in Yilmana Densa District, Northwest Ethiopia

Variables	Category	Frequency	Percent
Age	15–19	28	6.7
	20–29	202	48.1
	30–39	159	37.9
	40–49	31	7.4
Age of breastfed child	<6 months	110	26.2
	6–11 months	102	24.3
	12–24 months	208	49.5
Ethnicity	Amhara	420	100
Religion	Orthodox	414	98.6
	Muslim	6	1.4
Educational status	Unable to read and write	186	44.3
	Able to read and write	160	38.1
	Elementary school	50	11.9
	High school and above	24	5.7
Occupation	Farmer	369	87.9
	Merchant	29	6.9
	Others	22	6.2
Marital status	Unmarried	4	1.0
	Married	412	98
	Divorced	4	1.0
Family size	≤5	358	85.2
	>5	62	14.8
Landholding size	<1 hectare	112	26.7
	≥1 hectare	265	63.1
Monthly income	<1600 ETB	146	34.8
	≥1600 ETB	274	65.2

### 3.2 Underlying factors of undernutrition among lactating mothers

Household food security status, maternal care and health services, and household environment like water, sanitation, and hygiene are considered the main underlying causes of undernutrition and these all were assessed in this study. Therefore, household food security status was measured by household food insecurity access scale, and almost one‐quarter of the study respondents (24.5%) were found to be food insecure. This finding also revealed that the majority (83.3%) of the participants had antenatal follow‐up but their postnatal follow‐up is very low compared to the former one; similarly, this result showed that high percentage (83.1%, 90%) of the respondents were using family planning currently and gave birth at a health facility for their last child, respectively. As shown in Table 2, the majority (67.9%) of the study participants had **≤**2 children preceding the survey with a mean of 2.17 ± 1.73 number of children. Average birth‐to‐pregnancy interval history was assessed for the mothers who had at least two previous births; therefore, their birth‐to‐pregnancy interval was found to be 3.07 ± 0.870 years. And also more than half (51.9%) of the respondents have not received any nutrition information from health extension workers and other experts during pregnancy or lactation period. Furthermore, this study indicated that about 42.9% of the study participants did not have their own latrine/toilet or have no access to it; in addition, around 18% of the participants have no access to drinking tape/pipe water and hence they were using other sources of water like spring water, river water, and dug water (Table 2).

**TABLE 2:** Underlying factors of undernutrition among lactating mothers in Yilmana Densa District, Northwest Ethiopia

Underlying factors	Indicators	Category	Frequency	Percent
Food access	Household food security status	Food secure	317	75.5
		Food insecure	103	24.5
Maternal care and health services	Current family planning use	No	71	16.9
		Yes	349	83.1
	Number of parity	**≤**2	285	67.9
		>2	135	32.1
	Birth interval	**≤**2 years	71	27.1
		>2 years	191	72.9
	Place of delivery	Home	42	10.0
		Health institution	378	90.0
	ANC visit	No visit	70	16.7
		<4 visits	266	63.3
		≥4 visits	84	20.0
	PNC visit	No	266	63.3
		Yes	154	36.7
	Nutrition information	No	218	51.9
		Yes	202	48.1
Household environment [water, sanitation, and hygiene]	Access to safe drinking water [pipe water]	No	76	18.1
		Yes	344	81.9
	Toilet access	No	180	42.9
		Yes	240	57.1

### 3.3 Immediate factors of undernutrition among lactating mothers

Different evidence showed that nutritional status of an individual is directly/immediately affected by their quality and quantity of dietary intake as well as by their health status/disease experience. As per this argument, dietary diversity score of lactating mothers was calculated by summing up all number of food groups consumed by individual mothers over the 24‐h recall period; therefore, this study showed that the majority of lactating mothers (58.1%) have dietary diversity score of below the mean which is below four food groups with the mean dietary diversity score of 3.70 ± 1.148. Similarly, about one‐quarter (25.5%) of study participants were not taking any extra meals during their lactation period; and large proportion of the respondents (89% and 67.1%) did not take vitamin‐A‐rich fruits and vegetables and animal‐source foods during their lactation period, respectively; but all of the lactating mothers consumed plant‐based foods in the last 24‐h (Table 3). As shown in Table 3, about 33% of lactating mothers had eaten milk, egg, flesh meat, organ meat, or in combination within the 24‐h periods. And also only 12.6% of the study participants had a history of disease within the last 2 weeks prior to the survey, including malaria, typhoid fever, diarrhea, pneumonia, and gastric.

**TABLE 3:** Immediate factors of undernutrition among lactating mothers in Yilmana Densa District, Northwest Ethiopia

Immediate factors		Category	Frequency	Percent
Dietary intake	Women dietary diversity	<4 food groups	244	58.1
		≥4 food groups	176	41.9
	Frequency of meals per day	<3 meals/day	58	13.8
		≥3 meals/day	362	86.2
	Extra meal during lactation	No	107	25.5
		Yes	313	74.5
	Animal source food intake	No	282	67.1
		Yes	138	32.9
	Plant‐based food intake	No	0	0
		Yes	100	100
	Vitamin A source fruits and vegetables intake	No	375	89.3
		Yes	45	10.7
Disease	Sickness/disease	No	367	87.4
		Yes	53	12.6

### 3.4 Food groups consumed by lactating mothers in the last 24 h preceding the survey

**FIGURE 2:**
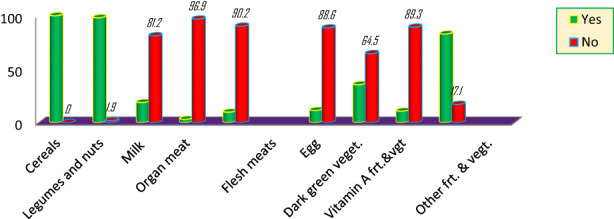
Food groups consumed by lactating mothers in the last 24 h preceding the survey in percentage.

All of the participants consumed cereal‐based foods mainly prepared from maize, teff, and millet; similarly, almost all (98.1%) participating mothers consumed legumes and nuts. As shown in Figure 2, the respondents of this study have a very less experience of consuming animal‐source foods and fruits and vegetables, only 3.1%, 9.8%, 11.4%, and 10.7% of participants consumed organ meat, flesh meat, egg, and vitamin‐A‐rich fruits and vegetables, respectively. Therefore, this result may reflect the vulnerability of lactating mothers to different micronutrient deficiencies due to inadequate intake of the aforementioned food groups. In contrary, cereals, legumes, and other fruits and vegetables mainly onion were the common food groups that most of the lactating mothers were taking in the last 24 h preceding the survey.

### 3.5 Anthropometry status of lactating mothers

Height in centimeters and weight in a kilogram of study participants were taken, and BMI was found to be 19.81 (±2.06). Among the total respondents, only 6.4% and 0.5% weighed less than 45 kg (underweight) and height less than 145 cm (stunted), respectively. Based on the BMI measurement, the prevalence of undernutrition among lactating mothers was found to be 22.6% (Table 4).

**TABLE 4:** Anthropometry measurement and nutritional status of lactating mothers in Yilmana Densa District, Northwest Ethiopia

Measurement	Category	Frequency	Percent
Height	<145 cm	2	0.5
	**≥**145 cm	418	99.5
Weight	<45 kg	27	6.4
	**≥**45 kg	393	93.6
Undernutrition [BMI]	No	325	77.4
	Yes	95	22.6

### 3.6 Nutritional status of lactating mothers determined by BMI measurements

**FIGURE 3:**
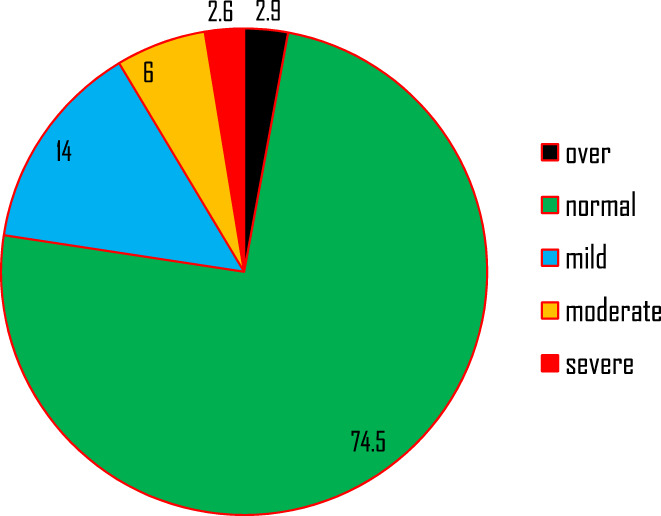
Nutritional status of lactating mothers by percentage in Yilmana Densa District Northwest Ethiopia.

Based on the BMI measurement, the prevalence of undernutrition among lactating mothers in the study area was 22.6%, among whom 2.6% were severely malnourished. Almost three of four lactating mothers had normal weight, whereas the rest (2.9%) of them were found to be overweight (Figure 3).

### 3.7 Factors associated with undernutrition among lactating mothers

Three categories of factors, mainly basic factors, underlying factors, and immediate factors, were identified to examine their association with undernutrition. Multivariable logistic regression analysis, household income, food security, dietary diversity, frequency of meals, potable water/pipe water accessibility, and latrine use were significantly associated with undernutrition, whereas age, family size, current use of family planning methods, place of delivery, nutrition information/counseling, ANC follow‐up, and any illness in the past 2 weeks were not significantly associated with undernutrition. Before running the model, model fitness and multicollinearity test were checked. The model output in Table 5 indicated that the odds of undernutrition among lactating mothers who had an average monthly household income of less than <1600 ETB were 3.5 times more likely to be undernourished than those who had greater monthly income (AOR = 3.51, 95%CI = 1.46–17.68). Similarly, lactating mothers who had no access to potable water/pipe water were seven times more likely to be undernourished than lactating mothers who have access to potable water/pipe water (AOR = 7.0, 95%CI = 1.05–46.85). The result of this study also revealed that the odds of undernutrition were almost six times higher in lactating mothers who use latrine as compared to their counterparts (AOR = 5.76, 95%CI = 1.36–24.43). And also, food‐insecure lactating mothers were 13 times more likely to be undernourished than lactating mothers who were food secure (AOR = 12.91, 95%CI = 2.85–58.43). Similarly, lactating women who had dietary diversity score of less than 4 food groups were nine times more likely to be undernourished than those who have diversity score of ≥4 food groups (AOR = 9.06, 95%CI = 3.74–22.8). Also, lactating women who ate less than three times per day were 14 times more likely to be undernourished as compared to those who ate more than or equal to three times per day (AOR: 14.19, 95% CI = 2.13–94.44) (Table 5).

**TABLE 5:** Multivariable logistic regression analysis output on factors associated with undernutrition of lactating mothers

Variable	Category	Undernutrition		COR (95%CI)	AOR (95%CI)	*p*‐Value
		Yes Freq. (%)	No Freq. (%)			
Age	15–19	8 (28.6)	20 (71.4)	3.733 (.88–15.85)	3.58 (.07–173.90)	.520
	20–29	50 (24.8)	152 (75.2)	3.070 (.90–10.53)	1.24 (.06–26.20)	.890
	30–39	34 (21.4)	125 (78.6)	2.539 (.738.86)	2.04 (.14–29.66)	.603
	40–49	3 (9.7)	28 (90.3)	1	1	
Family size	≤ 5	85 (23.7)	273 (76.3)	.62 (.30–1.27)	.69 (.072–6.624)	.747
	>5	10 (16.1)	52 (83.9)	1	1	
Household income	<1600 Birr	86 (57.3)	64 (42.7)	38.97 (18.61–81.6)	3.51 (1.46–17.68)	.001**
	≥1600 Birr	9 (3.3)	261 (96.7)	1	1	
Current FP use	No	21 (29.6)	50 (70.4)	1.56 (.88–2.76)	5.50 (.80–37.643)	.082
	Yes	74 (21.2)	275 (78.8)	1	1	
Place of delivery	Home	16 (38.1)	26 (61.9)	2.33 (1.91–4.55)	.27 (.035–2.00)	.198
	Health insti.	79 (20.9)	299 (79.1)	1	1	
ANC follow‐up	No	60 (84.5)	11 (15.5)	.020 (.01–.04)	7.179 (.85–60.84)	.071
	Yes	35 (10)	314 (90)	1	1	
Potable water/pipe water availability	No	31 (40.8)	45 (81.4)	3.3 (1.9–5.7)	7.0 (1.05–46.85)	.045*
	Yes	64 (18.6)	280 (81.4)	1	1	
Have latrine	No	85 (47.2)	95 (52.8)	20.58 (10.25–41.3)	5.76 (1.36–24.43)	.018*
	Yes	10 (4.2)	230 (95.8)	1	1	
Nutrition counseling	No	82 (36.6)	142 (63.4)	1	1	
	Yes	13 (6.6)	183 (93.4)	8.13 (4.35–15.18)	.81 (.162–4.033)	.796
Dietary diversity status	≥4 food groups	2 (.80)	242 (99.2)	1	1	
	<4 food groups	93 (52.8)	83 (47.2)	135.6 (32.7–562.4)	9.06 (3.74–22.8)	.001**
Food security status	Food insecure	80 (77.7)	23 (22.3)	70.03 (34.93–140.4)	12.91 (2.85–58.43)	.001**
	Food secure	15 (4.7)	302 (95.3)	1	1	
Sickness/illness	No	71 (19.3)	296 (80.7)	1	1	
	Yes	24 (45.3)	29 (54.7)	3.45 (1.89–6.29)	5.76 (.88–37.79)	.068
Frequency of meals	<3 meals	48 (82.8)	10 (17.2)	32.17 (15.24–67.9)	14.19 (2.13–94.44)	.006**
	≥3 meals	47 (13)	315 (87)	1	1	

*, **Stands for significance level at 5% and 1% probability.

## 4 DISCUSSION

The overall prevalence of undernutrition among lactating mothers in the study area was 22.6%, which is relatively consistent with Ethiopian Demographic and Health Survey National findings (22%) (CSA, 2016) and rural Ambo, Ethiopia (21.5%) (Black et al., 2008). However, this finding was higher than Arbaminch Zuria district, Southern Ethiopia (17.4%), Adama district of Oromia region (19.5%) (Abeya et al., 2018; Tikuye et al., 2018), the lowland part of Raya (17.5%) (Ismael et al., 2017), 19.3% of Jammu women and 10% of Kashmir women, India (Khan et al., 2012), and Ibadan, Nigeria (5%) (Sanusi & Falana, 2007). Probably it may be due to seasonal variation in which this study was conducted during the known hunger season in the study area that is July or the difference may be due to poor feeding practice of diversified food groups in which cereal‐based monotype food is common or it may be due to poor maternal nutrition intervention in the study area.

Household income was found to be one of the associated factors for undernutrition of lactating mothers, in which lactating mothers who got monthly income of greater than or equal to 1600 ETB were less likely to be undernourished than those lactating mothers who got less income. The probable reason may be that good household economic status may increase the capability of the mother to access adequate food and health services which in turn can improve the nutritional status of mothers. Therefore, it is justified that household income is a basic factor that may increase the purchasing power of mothers for goods and services, which ultimately contribute to improvements in their nutritional status. This finding is in line with the study conducted on lactating mothers at Nekemet and the study conducted on maternal and child nutrition in Ethiopia (Girma & Genebo, 2002; World Health Organization (WHO), 2009).

Food insecurity is also a major challenge for Ethiopia, especially women and children are very vulnerable groups. In this study, women from food‐secure households had a better nutritional status compared to women from food‐insecure households. The possible explanation for this could be that if there is a shortage of food supply and accessibility in the household, their meal quality and quantity is decreased and ultimately their dietary intake is affected, especially women; as a coping strategy for food shortage most of the time women/mothers are forced to eat less so as to feed the family. Similar finding is reported in the study conducted in Arbaminch Zuria district, Southern Ethiopia (Tikuye et al., 2018). However, a study conducted in the Raya district, Northern Ethiopia, reported that food security status did not associate with undernutrition (Ismael et al., 2017). This may be due to inappropriate measurement of food security status at the household level or it may be due to different socioeconomic backgrounds (almost all are food secure in Raya) of the study participants in the two study areas or it may be due to the variation of study period in which in this study the data were collected in more vulnerable month of the year for food insecurity. Dietary diversity score of the mother was found to be associated with undernutrition and this finding revealed that study participants who had consumed <4 food groups were more likely to be undernourished than those who had consumed ≥4 food groups. The probable reason may be that the more the mother consumed diversified food, the more likely she is to get quality and adequate nutrients including essential micronutrients which can improve the nutritional status of lactating mothers. This finding is supported by the study in Dedo and Seqa Chekorsa district, Jimma zone (Alemayehu et al., 2015), and study conducted in Hadiya zone, Southern Ethiopia (Abebe et al., 2019).

The unhealthy household environment also has significant association with maternal undernutrition, i.e., inadequate accessibility of pipe water and latrine contributes to undernutrition of lactating mothers in the study area. Based on the finding of this study, the prevalence of undernutrition among lactating mothers with no potable/pipe water source and with no toilet facility was significantly higher as compared with those who have the accessibility (AOR = 7 and 5.76), respectively. It may be due to unhealthy household environment, mainly unclean drinking water and poor toilet facility, which might cause different illnesses that affects their nutritional status immediately by loss of appetite and absorption of nutrients. This finding is consistent with the study finding conducted on determinants of maternal and child nutrition in Ethiopia and Kenya (Girma & Genebo, 2002).

The number of meals consumed by lactating mothers has a direct impact on their nutritional status and therefore, lactating mothers who ate less than three meals per day were more likely to be undernourished than those mothers who ate three or more meals per day. This finding is supported by a study conducted in Raya district, Northern Ethiopia, and Hadiya zone, Southern Ethiopia (Abebe et al., 2019; Ismael et al., 2017). The probable reason may be the improvement in calorie intake due to the increment in frequency of meal intake in a day.

## 5 CONCLUSION AND RECOMMENDATION

This study found that the prevalence of undernutrition in rural Yilmana Densa District was high, which was that more than one‐fifth of lactating mothers are undernourished. This study also identified that household income, food security status, dietary diversity, number of meals, and accessibility of potable/pipe water and latrine facility were factors significantly associated with undernutrition among lactating mothers in the study area. Therefore, to alleviate this problem, there should be strengthening of intersectoral collaboration within the agricultural sector to enhance production in advance and at the end of the day to improve household income, food security, and dietary diversity nutrition‐sensitive agriculture. Furthermore, there should be strengthening of social and behavioral change communication on the consumption of locally available diversified foods including animal source foods and fruits and vegetables by advocating nutrition‐sensitive agriculture; similarly, hygiene and sanitation coverage and utilization intervention work for rural community are very important to improve lactating mothers' nutritional status.

## FUNDING INFORMATION

No funding was obtained for this study.

## CONFLICT OF INTEREST

The authors declare that there are no competing interests.

## ETHICS APPROVAL AND CONSENT TO PARTICIPATE

Ethical clearance was obtained from the ethical clearance review committee of the Debre Markos University, College of health sciences. The purpose of the study was explained to the study participants and a written informed consent was taken from participants to confirm whether they were willing to participate or not. Respondents were informed that they can refuse or discontinue participation at any time they want, and they were also informed that they can ask anything about the study. To ensure confidentiality, the names of the study subjects were not written on the questionnaire and not shared with anyone else.

## Data Availability

The datasets used during the current study are available from the corresponding author upon reasonable request.
